# Case Report: Monitoring Vancomycin Concentrations and Pharmacokinetic Parameters in Continuous Veno-Venous Hemofiltration Patients to Guide Individualized Dosage Regimens: A Case Analysis

**DOI:** 10.3389/fphar.2021.763575

**Published:** 2021-12-09

**Authors:** Jihui Chen, Xiaohui Huang, Zhiyan Lin, Chao Li, Haoshu Ding, Junming Du, Lixia Li

**Affiliations:** ^1^ Department of Pharmacy, Xinhua Hospital, School of Medicine, Shanghai Jiao Tong University, Shanghai, China; ^2^ Department of Anesthesiology and SICU, Xinhua Hospital, School of Medicine, Shanghai Jiao Tong University, Shanghai, China

**Keywords:** vancomycin, continuous renal replacement therapy, therapeutic drug monitoring, area under the curve, kidney failure

## Abstract

There are limited pharmacokinetic (PK) studies on vancomycin in patients treated with continuous renal replacement therapy (CRRT), and the results have been inconsistent. Because of individual differences, proposing a definite recommendation for the clinical regimen is not possible. Rapidly reaching target vancomycin concentrations will facilitate effective treatment for critically ill patients treated with CRRT. In this study, to understand the dynamic change in drug clearance rates *in vivo*, analyze the effect of PK changes on drug concentrations, and recommend loading and maintenance dosage regimens, we monitored the blood concentrations of vancomycin and calculated the area under the curve in two critically ill patients treated with vancomycin and continuous veno-venous hemofiltration (CVVH). On the basis of real-time therapeutic drug monitoring results and PK parameters, an individualized vancomycin regimen was developed for patients with CVVH. Good clinical efficacy was achieved, which provided support and reference for empirical vancomycin therapy in these patients.

## Introduction

Vancomycin is mainly used for severe Gram-positive infections, especially those caused by methicillin-resistant *Staphylococcus aureus*, methicillin-resistant *Staphylococcus epidermis*, and *Enterococcus*. Approximately 90% of vancomycin is eliminated by the kidneys in its original form, therefore in patients with renal insufficiency, therapeutic agents may show a prolonged half-life and higher area under the curve (AUC) due to reduced drug clearance, leading to severe toxic reactions (Sun et al., 2015). While the use of CRRT in patients with acute renal failure is widely accepted (Prowle and Bellomo, 2010; Fayad et al., 2018). CRRT has also been used to remove inflammatory mediators in sepsis patients in surgical intensive care unit, however it also affects the metabolism and excretion of vancomycin (Petejova et al., 2014). Several factors influence the plasma drug concentration during CRRT by altering drug volume of distribution, drug metabolism, and drug elimination (Rybak et al., 2009).

Continuous veno-venous hemofiltration (CVVH) is a commonly used clinical treatment that mainly removes large- and medium-sized molecules from the body *via* convection. Vancomycin is a medium-sized substance with a molecular weight of 1,485.74 Da and is eliminated through semipermeable membranes. CRRT increases the clearance of free vancomycin, reducing the blood concentration in patients to below the treatment range (Blot et al., 2014). At present, the recommended dosage regimen of vancomycin during CRRT does not consider the individual factors of patients or the effects of the CRRT parameter setting. Therefore, it is challenging to reach and maintain an effective treatment concentration in a short time. In this case, the frequency of drug concentration monitoring should be increased to ensure effective treatment with vancomycin (Omrani et al., 2015). In this study, two patients with severe infection who received CVVH were included. Through regular monitoring of blood concentrations and individual dose adjustments, steady-state vancomycin concentrations were rapidly reached, thereby achieving effective treatment. This report provides a theoretical basis for the design of an individualized dosage regimen.

### Case Presentation

**FIGURE 1:**
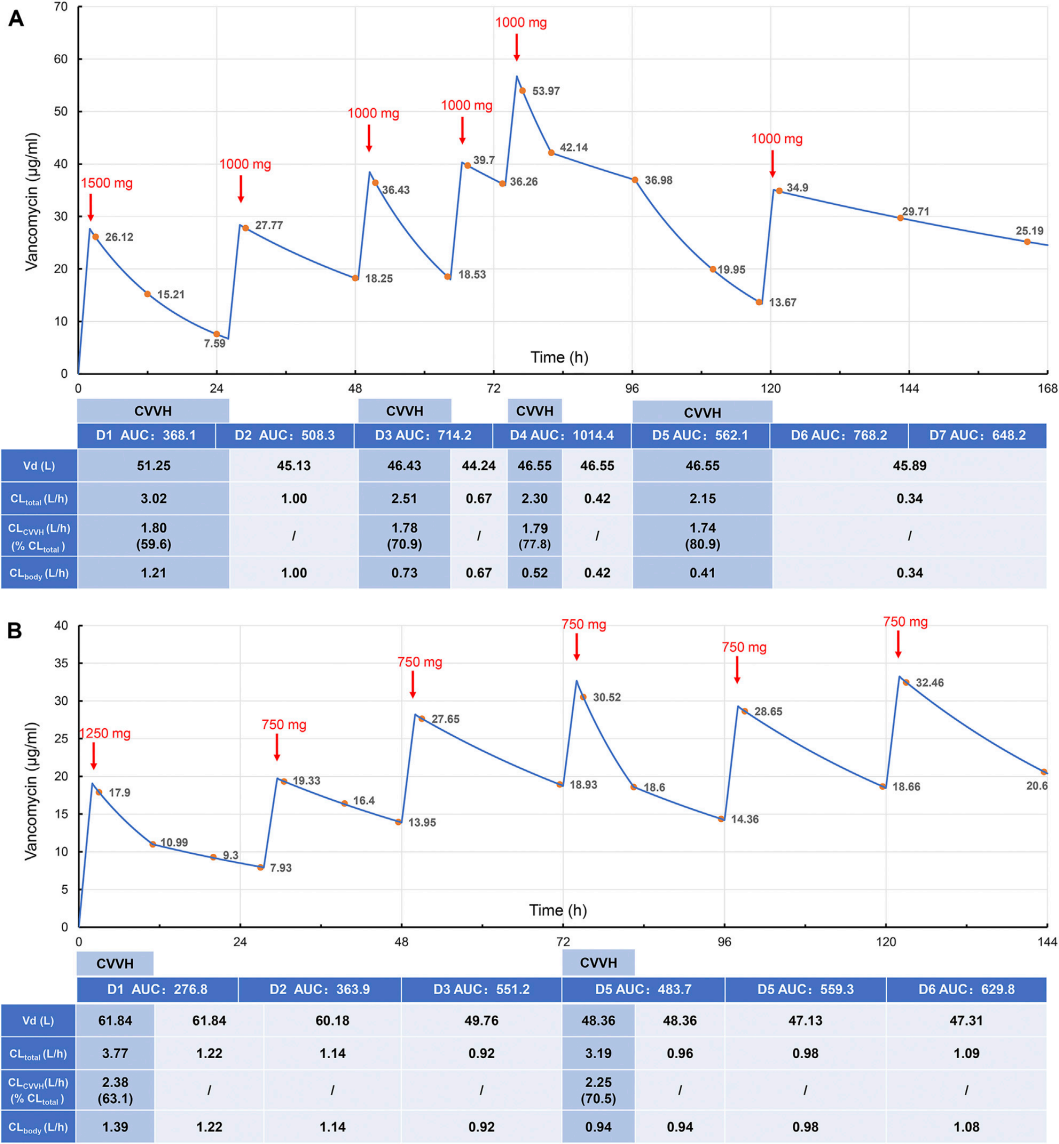
Vancomycin plasma concentration-time curve and pharmacokinetic parameters in case 1 **(A)** and case 2 **(B)**. The red arrows indicate the end time of intravenous administration, and the upper numbers indicate the doses. The yellow dots indicate the detected plasma concentrations of vancomycin, and the blue line is the fitted plasma concentration-time curve. The table shows the estimated vancomycin Vd, total clearance, CVVH clearance, endogenous clearance, and AUC of case 1 and case 2 for the corresponding time periods. AUC, area under the time-concentration curve over 24 h, mgh/L; Vd, volume of distribution; CL_total_, total vancomycin clearance; CL_CVVH_, CVVH vancomycin clearance; CL_body_, endogenous vancomycin clearance.

In this study, both patients were treated with CRRT (CVVH mode); the parameters are shown in Table 1. For the vancomycin regimen, the loading dosage of 25 mg/kg was administered intravenously for 2 h, and the plasma concentration was monitored at 1, 12, and 24 h post-administration. Then, vancomycin was administered at 15 mg/kg daily with 2-h infusion and the peak concentration was measured 1 h after administration. The regimen was adjusted according to the clinical situation and therapeutic drug monitoring (TDM) results. In addition, the drug concentration in the ultrafiltrate was determined to calculate the sieving coefficient (sieving coefficient, Sc, the ratio of drug concentration in the ultrafiltrate to plasma). The basic information and treatment results is shown in Tables 2, 3, and Vancomycin plasma concentration-time curve and pharmacokinetic parameters are shown in Figure 1.

**TABLE 1:** Parameters of CVVH during treatment with vancomycin.

Date	Duration of CVVH (h)	Dose of CVVH (ml/kg/h)	Pre-filter replacement fluid rate (ml/h)	Post-filter replacement fluid rate (ml/h)	Blood flow rate (ml/min)	Ultrafiltrate flow rate (ml/h)	Sieving coefficient
Case 1							
D1-D2	28	32	1,000	1,000	180	100	0.91
D3	16.5	32	1,000	1,000	180	100	0.90
D4	10	32	1,000	1,000	150	100	0.91
D5-D6	25	31	1,000	1,000	180	50	0.90
Case 2							
D1	11	59	2000	1,500	180	100	0.81
D5	10	58	2000	2000	170	100	0.78

**TABLE 2:** Clinical and demographic characteristics of patients^a^.

Basic information	Case1	Case2
Age (years)	37	93
Gender	male	female
Weight (kg)	62	50
Initial admission department	urinary surgery	general surgery department
Clinical diagnosis	septic shock, total cystectomy with ileal bladder replacement, urinary retention with pyuria, chronic renal insufficiency stage V, neurogenic bladder	gallstone, acute attack of chronic cholecystitis, biliary pancreatitis, septic shock, MODS
Term of operation	Intra-pouch lithotripsy with open intra-pouch and neobioresection *via* output-channel	ERCP
Background diseases	More than 20 years after total bladder operation; Renal insufficiency	High blood pressure. diabetes
CVVH treatment days prior to vancomycin administration	3	2
Pathogenic bacteria	Drainage fluid: *Enterococcus avium*	Bile: *Enterococcus faecium*
Other pathogenic bacteria	Drainage fluid: *Escherichia coli*	Blood, bile: *Escherichia coli*
Combined withr antibiotics	Meropenem	Imipenem and cilastatin
MIC of vancomycin	1	1
Loading dose (mg)	1,500	1,250
Maintenance dose (mg)	1,000	750
Scr (µmol/L)	1,355.9	269.6
ALT (U/L)	33	436
ALP (U/L)	49	120
Total bilirubin (µmol/L)	3.0	88.5
Direct Bilirubin (µmol/L)	1.4	43.1
Albumin (g/L)	24.3	27.9
Urine volume (ml)	0	250
Temperature (°C)	39.1	38.9
Heart rate (times/min)	120–142	120–145
Respiratory rate (times/min)	34	22
Systolic pressure (mmHg)	75	128
Lactic acid (mmol/L)	3.9	3.8
Ventilator assisted ventilation	yes	yes
SOFA (score)	18	19
WBC (10^9^/L)	5.3	56.5
Neut (%)	94.8	98
PLT (10^9^/L)	93	25
HB (g/L)	95	124
RBC (10^12^/L)	3.36	3.74
CRP (mg/L)	>160	>160
PCT (ng/ml)	>100	>100

a Data were from patients on the first day of vancomycin administration (D1). MODS, multiple organ dysfunction syndrome; ERCP, endoscopic retrograde cholangiopancreatography; CVVH, continuous veno-venous hemofiltration; MIC, minimum inhibitory concentration; Scr, serum creatinine; ALT, alanine transaminase; ALP, alkaline phosphatase; SOFA, Sequential organ failure assessment; WBC, white blood count; Neut, neutrophils; PLT, platelet; HB, hemoglobin; RBC, red blood cell; CRP, C-reactive protein; PCT, procalcitonin.

**TABLE 3:** Laboratory tests and results of patients during SICU.

Date	WBC (10^9^/L)	Neut (%)	PLT (10^9^/L)	RBC (10^12^/L)	HB (g/L)	CRP (mg/L)	PCT (ng/ml)	T (°C)	Scr (µmol/L)	Albumin (g/L)
Case1										
D1	6.90	93.2	42	3.25	92	160	>100	37.8	182.6	24.3
D2	14.4	96.1	26	3.77	106	>160	—	37	268.2	—
D3	17.9	91.8	28	3.52	98	>160	80.22	36.2	263.3	23.2
D7	18.78	96.1	77	2.71	77	154	20.22	36.6	197.8	24.9
D9	24.9	93.0	193	2.86	80	—	9.69	36.5	459	28
D13	16.3	88.3	343	2.36	68	57	3.60	36.7	—	24.7
D20	14.40	75.2	444	2.31	66	—	—	36.5	533	—
D22	10.40	71.0	420	2.22	63	—	1.40	37.0	745.8	25.2
Case2										
D1	25.50	94.5	186	4.03	129	102	3.10	36.7	235	29.8
D2	11.90	95.0	76	3.51	102	97	1.95	36.0	134	27.5
D4	12.60	91.4	213	3.19	103	74	0.87	36.4	194	—
D5	15.30	91.0	185	2.54	81	88	0.51	38.0	127.2	—
D7	9.10	88.6	252	2.55	81	—	—	36.6	142	28.3
D14	7.40	80.9	210	2.31	74	—	—	36.5	136	29.5

WBC, white blood count; Neut, neutrophils; PLT, platelet; RBC, red blood cell; HB, hemoglobin; CRP, C-reactive protein; PCT, procalcitonin; T, temperature; Scr, serum creatinine.

Vancomycin concentrations were determined by high-performance liquid chromatography, as described previously (Huang et al., 2021). The linear range for the assay was 2.0–100 mg/L, the lower detection limit was 1 mg/L, and intra- and inter-day precision and accuracy values were both within 15%. The vancomycin CVVH clearance (CL_CVVH_) was calculated from ultrafiltrate flow rate, plasma flow rate, replacement fluid flow rate, and Sc using the formulas previously published (Choi et al., 2009). The total vancomycin clearance (CL_total_) and volume of distribution (Vd) was calculated using a one-compartment model with first-order elimination from the concentrations obtained consecutively (Sawchuk and Zaske, 1976). The endogenous drug clearance (CL_body_) was calculated by subtracting CL_CVVH_ from CL_total_. AUC was calculated based on the log-linear trapezoidal rule.

#### Case 1

The first case was that of a 37-year-old man. At admission, the patient was diagnosed with septic shock, previous total cystectomy with ileal bladder replacement, urinary retention with pyuria, chronic renal insufficiency stage V, and neurogenic bladder. In addition, the patient’s vital signs were unstable, and noradrenaline and phenylephrine were required to maintain blood pressure after fluid resuscitation. Blood culture analyses were negative. *Escherichia coli* [extended spectrum beta-lactamase (ESBL) +], *Enterococcus avium*, and *Enterococcus faecium* were detected in the drainage fluid of the ileostomy. The antibiotic regimen at admission was 1000 mg meropenem q12 h ivgtt combined with vancomycin (loading dose on D1 of 1500 mg and maintenance dose of 1000 mg on D2). CVVH was also administered. From D7, the patient’s condition tended to be stable, and the CVVH frequency was reduced to every other day. On D9, puncture and catheterization of left subphrenic effusions were conducted under the guidance of B-ultrasound. *Escherichia coli* (ESBL+) and *Enterococcus avium* were identified in the drainage fluid. The intra-abdominal infection improved and the function of organs other than the kidney returned to normal. On D22, the culture of drainage fluid was negative, thus vancomycin and meropenem were stopped. On D26, the patient was transferred out of the intensive care unit and resumed routine hemodialysis.

#### Case 2

The second case was that of a 93-year-old woman. Her diagnosis at admission included a gallstone, acute attack of chronic cholecystitis, biliary pancreatitis, septic shock, and multiple organ dysfunction syndrome. The surgeons performed endoscopic retrograde cholangiopancreatography to relieve the biliary obstruction. The growth of *Escherichia coli* (ESBL +) and *Enterococcus faecium* was observed in bile bacterial culture during ultrasound-guided cholecystostomy. Blood cultures were positive for *Escherichia coli* (ESBL +). Mechanical ventilation and CVVH were performed at admission. The initial antibiotic treatment regimen comprised 500 mg imipenem/cilastatin q12 h ivgtt and 1,250 mg vancomycin ivgtt. Starting on D2, the patient was administered 750 mg vancomycin QD ivgtt. On D5, the patient was treated with CVVH again because of oliguria and elevated creatinine. On D7, blood culture tests were negative, thus imipenem/cilastatin was switched to piperacillin-tazobactam, and vancomycin was continued. On D14, the drainage fluid culture was negative and the patient was transferred to the general surgery department for further treatment. On D21, antimicrobial treatment was discontinued.

## Discussion

Vancomycin has a small apparent volume of 0.2–1.25 L/kg and is hydrophilic and easily eliminated by CRRT. Plasma protein binding rates are 30–55%, with an average 18% reduction in patients with renal failure, which results in an increased concentration of free vancomycin in the blood and significantly increased vancomycin clearance during CRRT. In a prospective PK study of seven patients with severe illness, the vancomycin Sc was 0.71 (±0.13), and the clearance rate by CVVH was 0.73 (±0.21) L/h (Chaijamorn et al., 2011). In our study, the Sc values in Case 1 and Case 2 were approximately 0.8 and 0.9, respectively, similar to the values reported in previous studies (Petejova et al., 2014; Charoensareerat et al., 2019). Together, these results indicate that CVVH effectively clears vancomycin. This results in a substantially higher drug clearance rate in patients during the CRRT stage than in the non-CRRT stage. It is difficult to determine the dosage regimen for these patients, and frequent blood concentration tests are needed to develop an individualized dosage regimen. Furthermore, the drug clearance rate is directly related to the dose of CRRT. Calculating the CRRT drug clearance rate and estimating the blood drug concentration by PK analysis is an effective alternative method when the blood drug concentration cannot be detected. In our study, the two cases treated with CRRT were severely ill patients with renal failure and septic shock. By combining the real-time plasma concentrations and specific PK parameters, an individualized vancomycin dosage regimen was developed for critically ill patients, achieving good clinical treatment outcomes.

The reported vancomycin recommended dose varies. A recent study recommended (Li et al., 2020a) that the ultrafiltrate flow rate of CVVH should be 30–40 mg/kg/h with 400–650 mg vancomycin administered q12 h. For CRRT patients, when the minimum inhibitory concentration (MIC) is ≥1 mg/L, the highest vancomycin dose recommended is 1.5 g/d. The most recent study recommended a higher total dose of ≥2.75 g/d (Charoensareerat et al., 2019). Therefore, it is necessary to adjust the plan according to the blood concentration results (Li et al., 2020b). Recently, the AUC/MIC ratio has become the preferred PK target for vancomycin therapy. However, individualized dosing remains difficult for patients with renal dysfunction and irregular use of CRRT. *The Guidelines for Monitoring Vancomycin Therapeutic Drugs in China (2020)* (He et al., 2020) recommended maintaining the target trough concentration of vancomycin at 10–15 mg/L for adult patients with common infections. The recommended target range of the AUC_0_–_24 h_ is 400–650 mg·h/L. The PKs in patients receiving CRRT are complex and difficult to predict, and the clinical environment is highly heterogeneous between studies. As a result, there is currently no standardized vancomycin protocol for these patients. PK models are recommended for individualized dosing. This study adjusted individual vancomycin dosage regimens mainly by monitoring blood drug concentrations and calculating AUCs. It was found that the monitoring data fluctuated greatly due to several influencing factors. Therefore, it is of important clinical significance to explore the initial dosage regimen, maintenance regimen, and AUC safety range of vancomycin in CRRT patients.

An effective concentration of vancomycin within 24 h of administration and an accurate initial dose of vancomycin are particularly important to quickly reach the target concentration. Therefore, the first loading dose is essential. The increase in drug distribution in critically ill patients results in lower drug concentrations than in non-critically ill patients when the same dose is given (Song et al., 2015). Therefore, in severely ill patients, a loading dose of vancomycin is more conducive to reach PK target. The initial dose of vancomycin suggested for CRRT patients is 20–25 mg/kg (Hoff et al., 2020; Rybak et al., 2020), with a recommended loading dose of 20–35 mg/kg (Matsumoto et al., 2013; Rybak et al., 2020). However, loading doses of vancomycin are rarely administered because of concern for potential kidney injury. In a previous study, a vancomycin loading dose computer system was used to increase the mean initial dose and trough concentration levels without increasing nephrotoxicity (Chun et al., 2021). In our study, 25 mg/kg was given on D1 post-administration, and the concentration in Case 1 and Case 2 decreased gradually within 24 h. Although they had different durations of CVVH, their concentrations were both lower than 10 mg/L. In patients treated with CVVH, the total clearance rate primarily depends on the amount of residual drug cleared by the kidney and the amount cleared by CVVH. The clearance rates in Case 1 and Case 2 were similar, and the clearance rate for CVVH was significantly higher than that for the kidney, which indicates that the influence of CVVH on drug concentration was important for the rapid decline in drug concentration *in vivo* within 24 h. Therefore, the dose or frequency of vancomycin administration on the day of CVVH should be increased to ensure effective therapeutic concentrations are achieved.

The maintenance regimen needs to be adjusted according to daily monitoring results, which is an important strategy to ensure the efficacy and safety of vancomycin. Irregular CVVH treatment will increase the fluctuation in trough concentrations and AUCs. The initial loading dose in this study was 25 mg/kg. If CVVH was stopped the next day, a maintenance dose of 15 mg/kg QD ivgtt was given. If CVVH treatment was continued on the next day, the drug was administrated twice on the first day with a loading dose of 25 mg/kg and a maintenance dose of 15 mg/kg in an interval of 12 h. The aim is to reduce fluctuations in the blood drug concentration on D1 and ensure that the drug concentration and AUC are maintained within the target range for a longer time during the first 24 h.

For Case 1, the concentration of vancomycin fluctuated greatly due to the changes in the daily CVVH duration, ultrafiltrate flow rate, and critical pathological status. Thus, it was difficult to adjust the maintenance regimen. On the basis of the vancomycin concentrations and AUC, the regimen was adjusted to 1000 mg vancomycin q48 h, followed by 1000 mg q72 h to control the infection. No CRRT was performed during the late stage in Case 2, and a fixed maintenance dose was given with the drug concentration and AUC within the target range. Therefore, it is essential to regularly monitor the changes in blood drug concentrations in real-time and adjust the regimen individually according to the monitoring results.

To date, no study has reported the safety range of vancomycin AUC values in CRRT patients. The AUC monitoring range of patients in this study fluctuated greatly, ranging from 276.8 to 1,014.4 mg/h/L. Monitoring the AUC alone is not suitable for patients with unstable renal function, and it is more feasible to administer the drug after the trough concentration is reduced to 10–20 mg/L (Roberts and Lipman., 2009). Because the trough concentration cannot be obtained quickly by TDM, this study adjusted the individualized regimen for patients with unstable renal function by combining the changes in the AUC and trough concentration. To explore the target range of vancomycin AUC values as a reference for dosage adjustment in CRRT patients, a clinical study with a large sample size is needed.

In conclusion, this study preliminarily determined the optimal initial loading dose (25 mg/kg) and maintenance dose (15 mg/kg, QD ivgtt or q48 h ivgtt) for patients treated with CVVH and vancomycin, achieving good clinical outcomes. In the future, clinical studies with a large sample size are needed.

## Data Availability

The raw data supporting the conclusion of this article will be made available by the authors, without undue reservation.
